# Autophagy-Related MicroRNA: Tumor miR-125b and Thyroid Cancers

**DOI:** 10.3390/genes14030685

**Published:** 2023-03-09

**Authors:** Liudmila V. Spirina, Irina V. Kovaleva, Svetlana Yu. Chizhevskaya, Anastasiya V. Chebodaeva, Nataliya V. Tarasenko

**Affiliations:** 1 Ministry of Health of Russia, Siberian State Medical University, 634050 Tomsk, Russia; 2 Cancer Research Institute, Tomsk National Research Medical Center of the Russian Academy of Sciences, 634050 Tomsk, Russia; 3 Research Institute of Medical Genetics, Tomsk National Research Medical Center of the Russian Academy of Sciences, 634050 Tomsk, Russia

**Keywords:** papillary thyroid cancer, autophagy, hsa-miR-125b, tumor invasion, BRAFV600E, risk of recurrence

## Abstract

Background: Autophagy is a stress response mechanism that causes cellular components to degrade. Its defects were associated with multiple pathologies, including cancers. Thyroid cancer is known to be the most prevalent form of malignant neoplasm among endocrine tumors. The aim of the study was to seek and comprehensively explore the role of autophagy related genes and proteins play in thyroid cancers through bioinformatics analysis with their detection in the tissue samples. Methods: Bioinformatics analysis was performed to investigate autophagy related proteins and genes involvement in thyroid cancer progression. The experimental verification was done in cancer samples of one hundred and three patients with thyroid pathology included in the study. The miR-125blevel was detected by PCR in real time. Results and discussion: The bioinformatics analysis verified the miR-125b as a regulatory mechanism in autophagy. Its expression in patients with PTC was reduced by 6.75 times in cancer patients compared to the patients with benign tumors. The BRAFV600E mutations were associated with a decrease in hsa-miR-125b expression by 12.67 times compared to tumors with the wild-type gene. Conclusions: Our findings revealed involvement of the autophagy related proteins in cancer progression. The significant mechanisms of regulation are non-coding RNA sequences implicated in a variety of oncogenic processes. We found that miR-125b is a potential maker in thyroid cancer invasion, BRAV600E mutational status and risk of recurrence.

## 1. Introduction

Autophagy is a stress response mechanism that leads to the degradation of cellular components, the defects of which are associated with multiple conditions [1]. Mounting evidence reveals that autophagy is a factor in thyroid cancer. Modulating autophagy has become a prominent target for improving the monitoring of cancer patients [2]. The principal triggers that determine the induction of autophagy are starvation, reactive oxygen species, endoplasmic stress and hypoxia. There are multiple channels that modulate autophagy, such as AMPK and PI3K orchestrating the protein cascade (ULK1, ATG16L1, MAP1LC3B, PRKAA1, ATG12, ATG14, ATG5, mTOR and BECN1) [2].

Papillary thyroid cancer (PTC) is the most frequent differentiated tumor. The malignant neoplasms rate in the thyroid gland is increasing annually worldwide [3]. The molecular mechanisms underlying the progress of PTC are complex and diversified. A number of key mutations are associated with PTC progression, including tyrosine kinase RET1 translocation, RAF protein mutation (BRAFV600E) and telomerase mutation (TERT1) [4]. However, their role in PTC is relatively low compared to the multiplication of oncogenic signals such as epigenetic factors.

Differentiated thyroid cancer (DTC) shows a good prognosis. The MAPK (mitogen-activated protein kinase) and AKT/mTOR signaling pathways are the main molecular events in the aggressive behavior of cancer. Promising approaches with a variety of targets have been found to restore autophagy in preclinical studies. But the complete molecular mechanism underlying the poor prognosis based on autophagy impairments remain fuzzy [1].

Recent studies have revealed the role of non-coding RNA sequences, particularly microRNAs, in a variety of pathologies. They are important non-coding endogenous RNA sequences of 22 long nucleotides that can regulate up to 30% of all human genes [5]. Small RNAs are involved in the most cellular functions [2]. Autophagy-regulating miRNA studied in cancer is a challenge in malignancies understanding and in the search for anti-cancer therapy targets.

Enhanced expression of p-ULK1 may promote autophagy by phosphorylating Beclin-1 and activating lipid kinase VPS34 [6]. The 16L1 autophagy-related protein (ATG16L1) indicates the process of autophagosome creation as well as the membrane closure process [7]. The light chain 3B protein associated with microtubule 1A/1B (hereinafter referred to as LC3) is a protein that in humans is encoded by the gene MAP1LC3B. It is a core protein in the autophagy pathway [8].

Targets of rapamycin (TOR) kinase-containing protein complexes are main regulators of the autophagic pathway. The mammalian TOR (mTOR) kinase forms two autophagic protein complexes: mTORC1 and mTORC2 [7]. Various stress-causing signals, deprivation and energy deficiencies are induced by the AKT/mTOR and the AMPK pathway. It orchestrates cell growth-related events and protein biosynthesis. The process is initiated by the Class III phosphatidylinositol 3-kinase complex (PI3K), comprising the proteins PI3K VPS34 and VPS30, ATG14/Barkor, VPS15 and ATG6/BECN1 (Beclin 1). The lipid kinase activity of the PI3K complex is responsible for the accumulation of phosphatidylinositol molecules 3-phosphate (PI3P) on the membranes, including the external foliole of the endoplasmic reticulum (RE). Two ubiquitylation-type conjugation systems, ATG12-ATG5-ATG16 and ATG8 (MAP1LC3, or briefly LC3 in mammals), regulate the elongation and completion of autophagic membranes [9].

MicroRNAs are considered novel tools in autophagy modulation. Several ones were associated with the change in autophagy-related proteins that are playing a role at various steps of the autophagy flux [6].

Hsa-miR-125b is derived from miR-125b-1 and miR-125b-2. In turn, miR-125b-1 was obtained from a long non-coding RNA (lncRNA)–MIR100HG (miR-100/let-7a-2/miR-125b-1, chromosome 11), and miR-125b-2 was obtained from the miRNA cluster (miR -99a/let-7c/miR-125b-2, chromosome 21). miR-125 is a highly conserved miRNA throughout diverse species. It consists of three homologs: hsa-miR-125a, hsa-miR-125b-1 and hsa-miR-125-2. miR-125 targets a number of genes such as transcriptional, growth factors and various components of signaling cascades [10].

Dysregulation of hsa-miR-125b is associated with tumor progression. It is known that small non-coding sequences of RNA can be presented both as oncogenes and tumor suppressors [11]. The hsa-miR-125b role in the oncogenes is still unclear. It was shown that it reduced the PI3K, phospho-Akt, and phospho-mTOR expression in anaplastic thyroid cancer cells [12]. In addition, it was found that miR-125b affects glucose metabolism by changing the proliferative activity of the tumor [13] and also affects the growth factor receptors [14] and transcription factors (NF-kB) [15], determining the metastatic and invasive cancer potential [16]. An association between the miRNA’s rate and the BRAFV600E mutational status was found. It is a significant indicator of the aggressive PTC phenotype [17].

Wang et al. (2018) demonstrated a new mechanism in hsa-miR-125b influence on the Foxp3 transcriptional factor. This factor is able to stimulate autophagy, modifying the sensitivity to chemotherapy [16,18]. Autophagy is a unique self-degrading process affecting cancer progression and initiation.

We aimed to seek and comprehensively explore the role of autophagy related genes and proteins play in thyroid cancers through bioinformatics analysis with their detection in the tissue samples.

## 2. Materials and Methods

### 2.1. Protein-Protein Interaction (PPI) Analysis

The Search Tool for the Retrieval of Interacting Genes/Proteins (STRING) used to review all known and predicted associations between proteins. The interacting network of autophagy-related proteins with other associated proteins were visualized through the STRING database. The cutoff value of interaction score was set to 0.15. A Venn diagram was utilized to display the interactions.

### 2.2. MicroRNA and Genes Associations

For pathway enrichment analysis, both Kyoto Encyclopedia of Genes and Genomes (KEGG) pathway analysis and Gene Ontology (GO) enrichment analysis was performed to visualize the cellular pathways. The cutoff Q value and P value were set at 0.05 and 0.01, respectively. The DIANA miRpath and PANTHER databases were used for bioinformatics analysis. HumanTargetScan (ver. 8.0) and miRTarBase (ver. 9.0) were used to seek target genes for miR-125. To identify the transcription factors, Transmir base (version 2.0) was used (https://www.cuilab.cn/transmir; accessed on 27 January 2023).

### 2.3. Clinical and Experimental Tools

The study included one hundred and three patients with thyroid pathology treated at the Cancer Research Institute of the Tomsk National Research Medical Center: sixty-seven patients had PTC and thirty-six patients had benign thyroid pathology. The T1-2N0M0 stage detected in twenty-seven patients and T3-4N0-1M0 was detected in forty patients. Metastases in regional lymph nodes were presented in twenty-seven patients. According to the histological subtype of PTC, patients were divided into a follicular subtype group (12 patients) and a classical subtype group (55 patients). The BRAFV600E mutation was identified in 18 patients. According to the criteria of the American Thyroid Association (ATA, American Thyroid Association), patients were divided into recurrence risk groups as follows: low risk of thyroid cancer recurrence was observed in 23 patients, intermediate risk in 25 patients and high risk in 19 patients.

This work was approved by the Local Ethical Committee in the Cancer Research Institute of the Tomsk National Research Medical Center. All procedures were carried out in accordance with the Protocol of the Helsinki Declaration on Human Rights (1964). All patients included in the study signed an informed consent to participate in the study.

The material of the study was samples of tumor and unchanged tissue obtained during surgery. Samples were taken in points located at a distance of at least 1 cm from the tumor boundaries. The samples were frozen and stored at a temperature of −80 °C.

### 2.4. DNA Extraction

DNA was isolated using the FFPET DNA Extraction Kit (Biolink, Russia). To assess the amount of isolated DNA, its concentration was assessed on a NanoDrop-2000 spectrophotometer (Thermo Scientific, Waltham, MA, USA). The resulting DNA was used for real-time PCR.

### 2.5. BRAFV600E Mutation Analysis

The BRAFV600E mutation was determined using the Real-time-PCR-BRAF-V600E kit (Biolink, Novosibirsk, Russia), designed to detect the GTG→GGG point mutation in codon 600 of the BRAF gene. The analysis was carried out by real-time allele-specific PCR.

### 2.6. Quantitative PCR with Reverse Transcription in Real Time

The tumor samples were incubated in RNAlater solution (Ambion, Foster City, CA, USA) for 24 h at 4 °C and then stored at −80 °C. MicroRNA was extracted using an isolation Kit (Biolabmix, Novosibirsk, Russia). The quality of the isolated nucleic acids was carried out using capillary electrophoresis on a TapeStation device (Agilient Technologies, Santa Clara, CA, USA). The RIN ranged from 2.2–3.3.

RT-qPCR was performed according to [Pfaffl MW]. PCR was conducted in 25 μL reaction volumes containing 12.5 μL BioMaster HS-qPCR SYBR Blue (2×) (“Biolabmix” Russia) and 300 nanoM of each primer: hsa-miR-125b: Forward primer 5′-GGATTCCCTGAGACCCTAAC-3′, Reverse primer 5′-GTGCAGGGTCCGAGGT-3′, RT primer 5′GTCGTATCCAGTGTCAGGGTCCGAGGTATTCGCACTGGATACGACTCACAAG-3′; U6: Forward primer 5′-CTCGCTT CGGCAGCACATATACT-3′, Reverse primer 5′-ACGCTTCACGAATTTGCGTGTC-3′, RT primer 5′-AAAATATGGAACGCTTC ACGAATTTGG-3′. A pre-incubation at 95 °C for 10 min was performed to activate the Hot Start DNA polymerase and denature the DNA and was followed by 45 amplification cycles of 95 °C denaturation at 95 °C for 10 s, 60 °C annealing at 60 °C for 20 s (iCycler iQ™, BioRad, Berkeley, CA, USA). The fold changes were calculated by the ΔΔCt method (the total ΔΔCt = fold of cancerous/normal tissue gene level), using normal tissue. The RNU6 was used as the endogenous control.

Statistical analysis was performed using the Statistica 12.0 software package (Tulsa, OK, USA). Normal distribution check was conducted using the Kolmogorov-Smirnov test. The results of gene expression determination are presented as Me (Q1; Q3). The Mann-Whitney test was used to assess significant differences. Differences were considered significant when *p* < 0.05.

## 3. Results

### 3.1. Investigation of MicroRNAs Related to Autophagy

#### 3.1.1. Investigation of MicroRNAs Regulating Proteins Associated with Autophagy

**Figure 1:**
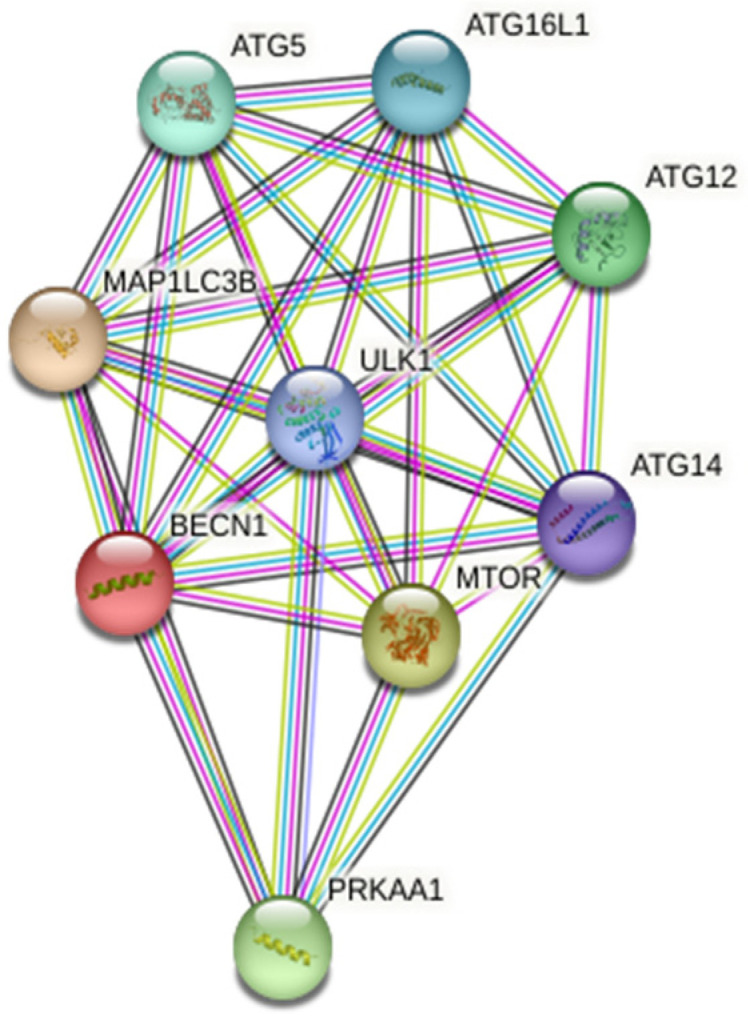
Protein-protein interactions between proteins associated with autophagy (https://string-db.org/; accessed on 27 January 2023). The edges colored purple reflect experimentally proven relationships, blue—relationships, information about which is obtained from supervised databases. Green edges reflect the neighborhood in the genome, blue—the joint occurrence, red—the fusion of genes. Light green ribs mean a joint mention of these proteins in Pub-Med Abstracts, black—co-expression, and light blue—homology. Note: Interactions: 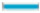
—from verified databases; 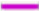
—experimentally determined interactions. Predicted interactions: 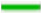
—neighborhood in the genome; 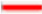
—gene fusion; 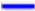
—occurrence. Other: 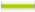
—joint mention of these proteins in PubMed Abstract; 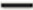
—co-expression; 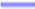
—homology.

Autophagy is a universal process of cellular viability as well as cancer cells. The key autophagy associated proteins are used to study the STRING database: ULK1, ATG16L1, MAP1LC3B, PRKAA1, ATG12, ATG14, ATG5, mTOR and BECN1 (Figure 1). The analysis showed the following associations between autophagy-associated proteins with multiple links. The edges colored purple reflect experimentally proven relationships, blue—relationships, information about which is obtained from supervised databases. Green edges reflect the neighborhood in the genome, blue—the joint occurrence, red—the fusion of genes. Light green ribs mean a joint mention of these proteins in Pub-Med Abstracts, black—co-expression, and light blue—homology. The found data highlighted the multiple interactions between the proteins.

The bioinformatics analysis in the DIANA microRNA database identified microRNAs regulating proteins associated with autophagy (Table S1). Twenty-two microRNAs were found for ATG12. ATG14 was regulated by the twenty-three microRNAs, ATG16L1 by seventy-five microRNAs, ATG5 by twenty-two microRNAs, BECN1 by forty microRNAs, MAP1LC3 by thirty microRNAs, ULK1 by one hundred six microRNAs, mTOR by thirty-three microRNAs, and PRKAA1 by two hundred sixteen microRNAs.

#### 3.1.2. MicroRNAs Associated with the Thyroid Cancers

At the next step, data on the microRNAs associated with the thyroid cancer and the proteins associated with autophagy were analyzed. Twenty-three microRNAs were identified with upregulated-expression and thirty-nine had oncosuppressor activity, which regulate all stages of oncogenesis (Table 1). However, among the targets associated with autophagy, only one microRNA is shown. Thus, miR-125b are able to regulate transcription and translation of the ATG5 protein, mediating the resistance to therapy in papillary thyroid cancer [19].

#### 3.1.3. Targets and Signaling Cascades Associated with MicroRNA 125

A bioinformatic analysis was performed to look for potential target genes and protein-protein interactions between their products (Table 2). Forty-four genes were found to be targets for the miR-125b. The detailed review showed the participation of the miR-125b in multiple processes: cell motility, immune response, DNA methylation, growth and transcriptional factors signal transduction. The MAPK and AKT/mTOR signaling cascades are found to be the key ones in autophagy [63]. PIK3CA, the initial component of the AKT/mTOR cascade [64]. CCDC6, protein 6 containing an antenna domain, interacts with CREB1 (reaction element binding protein cAMP 1) and suppresses its transcriptional activity [65].

**Figure 2:**
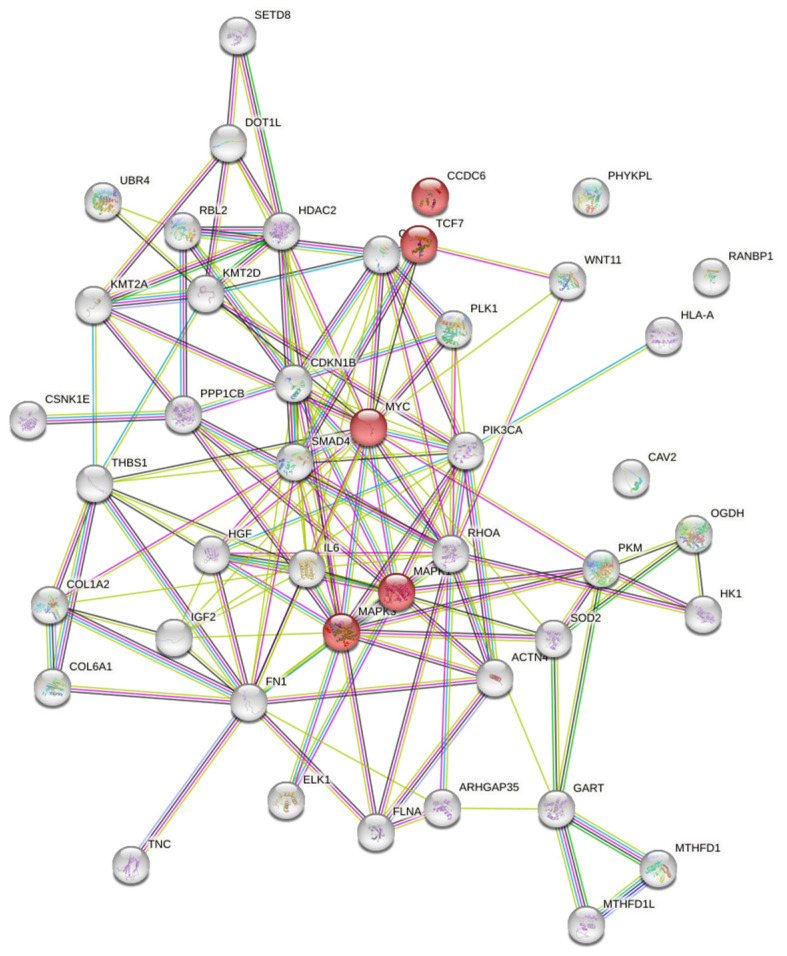
Protein-protein interactions between miR-125b target gene proteins (https://string-db.org/, accessed on 27 January 2023). Note: proteins associated with thyroid cancer are marked in red. Interactions: 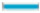
—from verified databases; 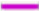
—experimentally determined interactions. Predicted interactions: 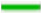
—neighborhood in the genome; 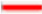
—gene fusion; 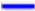
—occurrence. Other: 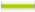
—joint mention of these proteins in PubMed Abstract; 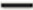
—co-expression; 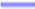
—homology.

The STRING database was used for the creation of the protein-protein interactions between the proteins of miR-125b associated genes (Figure 2). The complex scheme recovers only four target proteins that are involved in thyroid cancer progression.

KEGG pathway enrichment analysis revealed microRNA targets. They are components of the following signaling pathways: ECM-receptor interaction, proteoglycans in cancer, lysine degradation, adherent junction, viral carcinogenesis, thyroid cancer, focal adhesion, one carbon pool by folate, TGF-beta signaling pathway, colorectal cancers pathway (APC and Lynch syndrome), central carbon metabolism in cancer and FoxO signaling pathway. Of particular interest are the components involved in the molecular mechanisms of thyroid cancer development. Its main components are listed in Table 3.

The ECM-receptor interaction is essential in the cancer heterogeneity investigation [66]. Proteoglycans are molecules, responsible for cellular behavior modulation [67]. Cadherins and catenins are the central cell-cell adhesion molecules in adherens junctions (AJs). The significance of cadherin and catenin in cancer initiation and progression is well known [68]. The additional pathogenic mechanism is Lysine-specific demethylase 1 (LSD1) that stabilizes hypoxia-inducible factor 1α (HIF1α) to promote tumor progression [69]. The associations between viruses and cancer have been conducted in several studies and verified in meta-analyses [70]. Molecular alterations underlying PTC progression include deregulation of focal adhesion kinase (FAK), and PTC progression correlates with mRNA FAK-Del33 and pY397-FAK [71].

KEGG analysis revealed that one carbon pool by folate was connected to PTC pathogenesis [72]. A study by Xie et al. suggested that TGF-β1 triggers invasion and migration of PTC cells by inhibiting the expression of lncRNA-NEF [73].

We revealed gene and signaling components responsible for PTC manifestation. Lynch syndrome describing a familial cancer syndrome with four DNA mismatch repair genes mutations was found to be associated with PTC [74]. A high risk of familial adenomatous polyposis (FAP) was found in PTC patients [75].

PTC exhibits impaired energy metabolism, abnormal cell division, and more aggressive behavior through metabolic reprogramming and changes in the central carbon metabolism [76]. The FOXo signaling cascade is the important regulator of oncogenesis, which involves the mechanism of the RBM47/SNHG5/FOXO3 axis in PTC and also affects the triggering of autophagy [77].

**Figure 3:**
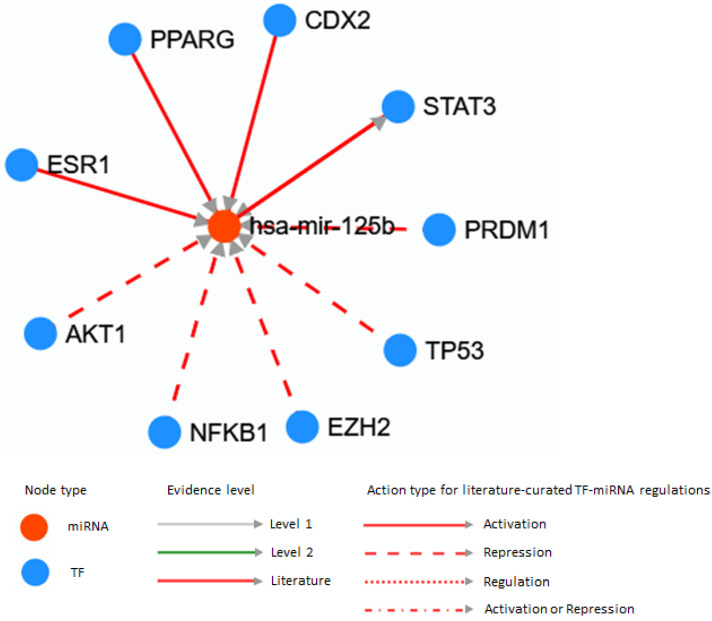
miR-125b and transcription factors associations (https://www.cuilab.cn/transmir; accessed on 27 January 2023). Note: level 1—regulation of TF-miRNA is associated with the analysis of a large number of microRNAs (theoretical connection); level 2—regulation of TF-miRNA is associated with the analysis of experimental data.

The association of microRNA 125b with transcription factors is shown in Figure 3. The association of hsa-mir-125b with STAT3, PRDM1, TP53, EZH2, NFkB1, AKT1, ERS1 and PPARG was revealed.

STAT3 transcriptional factor regulates PTC metastasis via glycolysis modification [78]. PRDM1 is a key target in Hashimoto thyroiditis [79]. TP53 is an oncosuppressor associated with metastatic PTC [80].

Recent studies revealed the EZH2 gene (histone methyltransferase) in modulating the cancer invasiveness and metastatic potential [81]. NF-κB induction is a key process in cancer progression and promotes NFKB1 [82]. The somatic mutations were identified in AKT genes and is associated with PTC risk [83].

Thyroid cancers are prevalent in women, indicating the sex hormone disorders as an essential risk factor [84]. The peroxisome proliferator receptor gamma (PPARγ) alteration associated with steroids transcriptional factors was detected only in follicular thyroid cancer and follicular adenoma [85].

### 3.2. MicroRNA 125b Expression in Thyroid Pathologies

The miRNA expression was reduced by 6.75 times in patients with PTC compared with the benign tumors. miRNAs are found to play a multifaceted role in oncogenesis. It is believed that they could be both oncoproteins and oncosuppressors, guiding the cancer progression potential [11].

Table 4 presents data on the microRNA expression in PTC. Thus, tumor size and regional lymph nodes involvement were not associated with the hsa-miR-125b level. However, in patients with the thyroid gland capsule invasion, hsa-miR-125-b expression increased 2.36 times compared to patients without signs of invasion.

The histological subtype of thyroid cancers was associated with miR-125b expression. A decrease of 37.0 times was found in the follicular subtype compared to tumors with the classical subtype. Additionally, in patients with positive BRAF V600E mutational status, a significant decrease in hsa-miR-125b levels by 12.67 times was shown compared to tumors without the mutation.

To assess the miR-125b contribution to the PTC relapse prediction, we studied the miRNA expression in groups according to the criteria of the American Thyroid Association (ATA, American Thyroid Association). In patients with an intermediate risk of recurrence, the miR 125-b expression was significantly increased by 107.09 and 11.78 times compared with the low- and high-risk groups, respetively.

## 4. Discussion

Autophagy is a universal process of the cellular viability as well as cancer cells. For nine key autophagy associated proteins (ULK1, ATG16L1, MAP1LC3B, PRKAA1, ATG12, ATG 14, ATG5, mTOR and BECN1), we found five hundred sixty-two microRNAs (in Supplements). Among them only sixty-nine are found to be correlated with PTC. However, among the targets associated with autophagy, only miR-125b is able to regulate transcription and translation of the ATG5 protein, mediating the resistance to therapy in PTC [19].

Non-coding RNA sequences, such as miRNAs, are essential for the autophagic flux modification, from upstream signaling pathways to later stages of autophagy. We found the theoretical and experimental verification of hsa-miR-125b implication in thyroid cancer progression. The revealed forty-four genes were found to be targets for miR-125b. KEEG data analysis and the DIANA microRNA investigation tool show that a variety of genes and transcriptional factors guided the PTC progression. But only few of them are under consideration in PTC research. We indicated four target proteins associated with PTC. They belong to the two signaling cascades that contribute to the cancer progression. MAPK1 and MAPK3 are the components of the rout that orchestrate the rapid cancer cell division and determine the aggressive cancer cells behavior [63]. PIK3CA activates AKT/mTOR signaling and operates the protein’s metabolism as a key mechanism of cells adaptation and survival [64]. The novel genetic marker is CCDC6, the components of the gene’s translocation in PTC [65]. It should be noted that the role of most molecular and genetic factors is still unknown, which causes the unpredictable pattern of behavior of tumor cells and leads to the low treatment response.

MiR-125b targets a number of genes such as components of signaling cascades and transcriptional factors [10]. Most of them are universal and highlight the variety of oncogenic processes: cell motility [66,67,68,69,70,71], epigenetic regulation [69] as well as metabolic reprogramming [72,76], viruses [70], immune response [73] and familial genetic disorders [74,75]. It was again mentioned that the FOXo signaling cascade participation in PTC oncogenesis as well as in autophagy initiation [77].

Transcriptional factor activation is the hidden inner mechanism in cancer progression. The association of hsa-mir-125b with STAT3, PRDM1, TP53, EZH2, NFkB1, AKT1, ERS1 and PPARG was revealed. The complex of factors represented the processes activated in PTC development [78,79,80,81,82,83,84,85]. They recover the mechanism of immune response, microenvironment modification, hormone receptors signal transduction, oxidative stress and etc.

Currently, the diagnostic application of microRNAs in human tumor diseases, as well as for recurrence detection after therapy, is widely discussed. Multidirectional changes in the miR-125b level in the PTC were revealed. The presented data indicate the miRNAs involvement in oncogenesis. There was a decrease in the hsa-miR-125b rate found in patients with PTC compared with benign pathology tissues. But the PTC classical subtype was associated with an increased level of expression.

It is known that the tumor invasion is the cancer progression, associated with the intracellular signaling, transcription and growth factors changes [12,37,65] and reflected in the aggressive tumor behavior. In addition, a high hsa-miR-125b expression was associated with the BRAF V600E status, which was disclosed in the results of the experiment on melanoma in cell culture [17]. The study showed an association between the cancer mutational status and hsa-miR-125b expression.

The tumor invasive potential may be associated with the autophagy, modulating the transformed cell behavior [18]. Activation of “cell self-digestion” is significant in the PTC progression, including the therapy effectiveness. It was found that miRNA expression differs depending on the disease recurrence risk. The maximum values of the expression were recorded for patients with an intermediate risk, which is probably of decisive importance, since tumors with an intermediate risk of relapses may have biological features associated with an aggressive phenotype. In previous research, the LC3B protein content, an autophagosome protein, was noted in this group of patients [86]. Some components of the autophagic machinery have important non-autophagic cellular functions. High MAP1LC3B levels could result from cancer progression and reflect the low sensitivity of anti-cancer therapy [87].

## 5. Conclusions

Five hundred and sixty-seven miRNAs associated with nine autophagy proteins (ULK1, ATG16L1, MAP1LC3B, PRKAA1, ATG12, ATG 14, ATG5, mTOR and BECN1) were identified, of which sixty-two are accompanied by thyroid cancer, and only miR-125b is associated with the autophagy modulation in this pathology.

The involvement of the non-coding RNA sequences remains unclear. It found the multiple genes and transcription factors used in oncogenesis. The concept of the paper was to find the pool of miRNAs associated with autophagy, and then identify the most powerful ones that regulate the oncogenesis in the thyroid gland. The interesting trend was again verified for the thyroid cancers, that determination of one isolated single pathway and route does not explain and predict cancer behavior. There should be found the molecular factors combination that are essential for the oncogenesis, providing new prominent targets in search for anti-cancer therapy.

Autophagy is a universal, powerful tool for thyroid cancer progression. Our findings revealed the hsa-miR-125b expression changes in thyroid cancer invasion, in BRAF V600E mutant tumors and in intermediate recurrence risk. High expression of hsa-miR-125b is a feature of the classical tumor subtype, as well as for tumors with invasion into the thyroid gland capsule. The data obtained indicate the role of miRNAs in the formation of the tumor invasive properties. The role of miRNAs is planned to be investigated further by a more comprehensive study.

## Figures and Tables

**Table 1:** List of microRNAs associated with thyroid cancer.

Change in Expression	MicroRNAs (Target/Regulator)	Reference
microRNAs upregulated in thyroid cancers	hsa-miR-146a-5p (TRAF and PML)	[20]
	hsa-miR-146b-5p (RARB)	[21]
	hsa-miR-146b-5p (DICER1)	[22]
	hsa-miR-221 (AXIN, BCL2, RUNX1, CCNE2)	[23]
	hsa-miR-221-3p	[24]
	let-7p	[25]
	miR-144 (AXIN, BCL2, RUNX1, CCNE2, mTOR)	[23,26]
	miR-146a/miR-146b (IRAK1)	[27]
	miR-146b	[28]
	miR-155	[29]
	miR-17-5p	[22]
	miR-181a (RB1)	[30]
	miR-181b	[29]
	miR-182 (TRIM8)	[31]
	miR-19a (PTEN, TSHr, Tg, TTF1 and Pax8, CDH1, an E-cadherin)	[32]
	miR-205 (VERG-A, ZEB1)	[33]
	miR-221 (RECK, TIMP3)	[34,35,36]
	miR-223 (APQ-1)	[37]
	miR-23a (PTEN)	[38]
	miR-340 (BMP4)	[39]
	miR-34a (AXIN, BCL2, RUNX1, CCNE2, AXL)	[23,26]
	miR-451a	[22]
	miR-9-3p (BLCAP)	[40]
microRNA as oncosupressor	miR-132-3p	[22]
	let-7a	[41]
	let-7b (HMGA2)	[41]
	miR-101	[36]
	miR-1179	[37]
	miR-125b (Foxo3, ATG5)	[24]
	miR-128 (SPHK1, Bmi-1, EGFR and E2 F3)	[42]
	miR-129 (MAL2)	[43]
	miR-130a	[12]
	miR-132 (FOXA2)	[44]
	miR-139b-5p (RICTOR, SMAD2/3 and HNRNPF)	[45]
	miR-144	[36]
	miR-144 (E2F8)	[46]
	miR-146a-5p	[22]
	miR-146b	[36]
	miR-148a	[24]
	miR-153-3p (RPS6KB1)	[47]
	miR-181b	[24]
	miR-194	[24]
	miR-199a-3p	[48]
	miR-203 (Survivin)	[49]
	miR-205 (VERG-A, ZEB1, YAP1)	[50]
	miR-21	[51]
	miR-212 (SIRT1)	[52]
	miR-217 (AKT3)	[53]
	miR-23a (CCNG1)	[54]
	miR-24	[24]
	miR-26b-5p (Gsk-3β and β-catenin)	[55]
	miR-30a	[24]
	miR-335-5p (ICAM-1)	[56]
	miR-34a	[26]
	miR-34a (MET, XIST)	[57]
	miR-424	[24]
	miR-429 (ZEB1)	[58]
	miR-451	[59]
	miR-577 (SphK2)	[60]
	miR-615	[24]
	miR-9 (BRAF)	[61]
	miR-let-7e (HMGB1)	[62]

**Table 2:** Target genes for microRNA 125b.

Target Gene	Description
*ACTN4*	Alpha-actinin-4;
*ARHGAP35*	Rho GTPase-activating protein 35;
*CAV2*	Caveolin-2;
*CCDC6*	Coiled-coil domain-containing protein 6
*CDK6*	Cyclin-dependent kinase 6;
*CDKN1B*	Cyclin-dependent kinase inhibitor 1B;
*COL1A2*	Collagen alpha-2(I) chain;
*COL6A1*	Collagen alpha-1(VI) chain;
*CSNK1E*	Casein kinase I isoform epsilon;
*DOT1L*	Histone-lysine N-methyltransferase;
*ELK1*	ETS domain-containing protein Elk-1;
*FLNA*	Filamin-A;
*FN1*	Fibronectin 1;
*GART*	Phosphoribosylamine--glycine ligase;
*HDAC2*	Histone deacetylase 1/2;
*HGF*	Hepatocyte growth factor;
*HK1*	Hexose kinase.
*HLA-A*	HLA class I histocompatibility antigen;
*IGF2*	Insulin-like growth factor II;
*IL6*	Interleukin-6;
*KMT2A*	[histone h3]-lysine4 n-trimethyltransferase;
*KMT2D*	Histone methyltransferase. Methylates ‘Lys-4’ of histone H3 (H3K4me).
*MAPK1*	Mitogen-activated protein kinase 1;
*MAPK3*	Mitogen-activated protein kinase 3;
*MTHFD1*	C-1-tetrahydrofolate synthase;
*MTHFD1L*	Monofunctional C1-tetrahydrofolate synthase;
*MYC*	Myc proto-oncogene protein;
*OGDH*	2-oxoglutarate dehydrogenase;
*PHYKPL*	5-phosphohydroxy-L-lysine phospho-lyase;
*PIK3CA*	Phosphatidylinositol 4,5-bisphosphate 3-kinase;
*PKM*	Pyruvate kinase m1/2; Pyruvate kinase PKM;
*PLK1*	Serine/threonine-protein kinase PLK1;
*PPP1CB*	Serine/threonine-protein phosphatase PP1-beta catalytic subunit;
*RANBP1*	Ran-specific GTPase-activating protein;
*RBL2*	Rb transcriptional corepressor like 2;
*RHOA*	Ras homolog gene family, member a;
*SETD8*	[histone h4]-lysine20 n-methyltransferase setd8;
*SMAD4*	Mothers against decapentaplegic homolog 4;
*SOD2*	Superoxide dismutase [Mn], mitochondrial;
*TCF7*	Transcription factor 7;
*THBS1*	Thrombospondin-1;
*TNC*	Tenascin;
*UBR4*	E3 ubiquitin-protein ligase UBR4;
*WNT11*	Wingless-type mmtv integration site family, member 11

**Table 3:** The signaling cascades and their components, associated with miR-125b.

Signaling Cascade	Components
ECM-receptor interaction	THBS1
	COL6A1
	COL1A2
	FN1
	TNC
	LAMA4
Proteoglycans in cancer	THBS1
	RHOA
	CAV2
	MYC
	IGF2
	FLNA
	MAPK3
	WNT11
	PIK3CA
	FN1
	HGF
	MAPK1
	ELK1
	PPP1CB
Lysine degradation	OGDH
	SETD8
	PHYKPL
	KMT2D
	DOT1L
	KMT2A
Adherens junction	PVRL2
	RHOA
	TJP1
	SMAD4
	MAPK3
	ACTN4
	EP300
	TCF7
	MAPK1
Viral carcinogenesis	RBL2
	RANBP1
	PKM
	CDKN1B
	RHOA
	CDK6
	HDAC2
	MAPK3
	ACTN4
	EP300
	PIK3CA
	MAPK1
	HLA-A
	UBR4
Thyroid cancer	MYC
	MAPK3
	TCF7
	MAPK1
	CCDC6
Focal adhesion	THBS1
	RHOA
	CAV2
	COL6A1
	ARHGAP35
	FLNA
	MAPK3
	COL1A2
	ACTN4
	PIK3CA
	FN1
	TNC
	HGF
	MAPK1
	ELK1
	PPP1CB
	LAMA4
One carbon pool by folate	MTHFD1L
	MTHFD1
	GART
TGF-beta signaling pathway	THBS1
	RHOA
	SMAD4
	MYC
	MAPK3
	EP300
	MAPK1
Colorectal cancer	RHOA
	SMAD4
	MYC
	MAPK3
	PIK3CA
	TCF7
	MAPK1
Central carbon metabolism in cancer	PKM
	MYC
	MAPK3
	PIK3CA
	HK1
	MAPK1
FoxO signaling pathway	RBL2
	CDKN1B
	SMAD4
	MAPK3
	CSNK1E
	EP300
	SOD2
	PIK3CA
	PLK1
	IL6
	MAPK1

**Table 4:** The miR-125b expression in tumor tissue in patients with thyroid pathology.

Indicator	Hsa-miR-125-b Expression Level (Relative Units)	
Patients with thyroid pathology	Benign thyroid pathology	6.75 (1.15; 18.38)
	PTC	1.00 (0.19; 4.59) #
Tumor size	T1-2N0M0	1.15 (0.07; 10.56)
	T3-4N0-1M0	1.00 (0.62; 3.03)
Regional metastases	T1-2N0M0	1.07 (0.14; 4.59)
	T3-4N1M0	0.81 (0.19; 18.38)
Invasion	−	1.00 (0.14; 3.03)
	+	2.63 (0.19; 24.25) *
Histological subtype	Classical subtype	1.11 (0.33; 10.56)
	Follicular subtype	0.03 (0; 0.14) ***
BRAFV600E mutational status	−	2.66 (0.87; 32.00)
	+	0.21 (0.01; 0.66) **
Risk of disease recurrence after treatment (ATA)	Low risk	0.11 (0.02; 1.24)
	Intermediate risk	11.78 (2.39; 28.13) ****
	High risk	1.00 (0.62; 1.52)

Note: #—significance of differences compared to patients with benign tumors, *p* < 0.05; *—significance of differences compared to patients with signs of tumor, *p* < 0.05; **—significance of differences compared to patients with follicular subtype of PTC, *p* < 0.05; ***—significance of differences compared to patients with BRAFV600E mutation, *p* < 0.05; ****—significance of differences compared with patients in group with low risk of recurrence after the treatment, *p* < 0.05.

## Data Availability

All the relevant data have been provided in the manuscript, and any supplementary datasets used and/or analyzed during the current study are available from the corresponding author on reasonable request.

## Supplementary Materials

The following supporting information can be downloaded at: https://www.mdpi.com/article/10.3390/genes14030685/s1, Table S1: List of microRNAs associated with the autophagy-related proteins.

## References

[1] Oh J.M., Ahn B.C. (2021). Molecular mechanisms of radioactive iodine refractoriness in differentiated thyroid cancer: Impaired sodium iodide symporter (NIS) expression owing to altered signaling pathway activity and intracellular localization of NIS. Theranostics.

[2] Holm T.M., Yeo S., Turner K.M., Guan J.L. (2022). Targeting Autophagy in Thyroid Cancer: EMT, Apoptosis, and Cancer Stem Cells. Front. Cell Dev. Biol..

[3] Jiang S., Huang Y., Li Y., Gu Q., Jiang C., Tao X., Sun J. (2022). Silencing FOXP2 reverses vemurafenib resistance in BRAFV600E mutant papillary thyroid cancer and melanoma cells. Endocrine.

[4] Kuo C.Y., Jhuang J.Y., Huang W.C., Cheng S.P. (2022). Aberrant Expression of Thymosin Beta-4 Correlates with Advanced Disease and BRAF V600E Mutation in Thyroid Cancer. J. Histochem. Cytochem..

[5] Bartel D.P. (2004). MicroRNAs: Genomics, biogenesis, mechanism, and function. Cell.

[6] Russell R.C., Tian Y., Yuan H., Park H.W., Chang Y.Y., Kim J., Kim H., Neufeld T.P., Dillin A., Guan K.L. (2013). ULK1 induces autophagy by phosphorylating Beclin-1 and activating VPS34 lipid kinase. Nat. Cell Biol..

[7] Lu C., Chen J., Xu H.G., Zhou X., He Q., Li Y.L., Jiang G., Shan Y., Xue B., Zhao R.X. (2014). MIR106B and MIR93 prevent removal of bacteria from epithelial cells by disrupting ATG16L1-mediated autophagy. Gastroenterology.

[8] Guo L., Stripay J.L., Zhang X., Collage R.D., Hulver M., Carchman E.H., Howell G.M., Zuckerbraun B.S., Lee J.S., Rosengart M.R. (2013). CaMKIα regulates AMP kinase-dependent, TORC-1-independent autophagy during lipopolysaccharide-induced acute lung neutrophilic inflammation. J. Immunol..

[9] Spirina L.V., Avgustinovich A.V., Afanas'ev S.G., Cheremisina O.V., Volkov M.Y., Choynzonov E.L., Gorbunov A.K., Usynin E.A. (2020). Molecular Mechanism of Resistance to Chemotherapy in Gastric Cancers, the Role of Autophagy. Curr. Drug Targets.

[10] Sun Y.M., Lin K.Y., Chen Y.Q. (2013). Diverse functions of miR-125 family in different cell contexts. J. Hematol. Oncol..

[11] Peng B., Theng P.Y., Le M.T.N. (2021). Essential functions of miR-125b in cancer. Cell Prolif..

[12] Bu Q., You F., Pan G., Yuan Q., Cui T., Hao L., Zhang J. (2017). MiR-125b inhibits anaplastic thyroid cancer cell migration and invasion by targeting PIK3CD. Biomed. Pharmacother..

[13] Zhang G., Zhou S., Yang Q., Liu F. (2020). MicroRNA-125b reduces glucose uptake in papillary thyroid carcinoma cells. Oncol. Lett..

[14] Lowery A.J., Miller N., McNeill R.E., Kerin M.J. (2008). MicroRNAs as prognostic indicators and therapeutic targets: Potential effect on breast cancer management. Clin. Cancer Res..

[15] Murphy A.J., Guyre P.M., Pioli P.A. (2010). Estradiol suppresses NF-kappa B activation through coordinated regulation of let-7a and miR-125b in primary human macrophages. J. Immunol..

[16] Ghafouri-Fard S., Shirvani-Farsani Z., Taheri M. (2020). The role of microRNAs in the pathogenesis of thyroid cancer. Noncoding RNA Res..

[17] Sánchez-Sendra B., González-Muñoz J.F., Pérez-Debén S., Monteagudo C. (2022). The Prognostic Value of miR-125b, miR-200c and miR-205 in Primary Cutaneous Malignant Melanoma Is Independent of BRAF Mutational Status. Cancers.

[18] Wang S., Wu J., Ren J., Vlantis A.C., Li M.Y., Liu S.Y.W., Ng E.K.W., Chan A.B.W., Luo D.C., Liu Z. (2018). MicroRNA-125b Interacts with Foxp3 to Induce Autophagy in Thyroid Cancer. Mol. Ther..

[19] Plantinga T.S., van de Vosse E., Huijbers A., Netea M.G., Joosten L.A., Smit J.W., Netea-Maier R.T. (2014). Role of genetic variants of autophagy genes in susceptibility for non-medullary thyroid cancer and patients outcome. PLoS ONE.

[20] Lin S., P. L., Wen D., Gao L., Liang L., Luo Y., Wei Y., Yu H., Yang H., Ma W. (2018). Expression level of miR-146b-5p via miRNA sequencing and its potential targets in papillary thyroid cancer. Int. J. Clin. Exp. Med..

[21] Czajka A.A., Wójcicka A., Kubiak A., Kotlarek M., Bakuła-Zalewska E., Koperski Ł., Wiechno W., Jażdżewski K. (2016). Family of microRNA-146 Regulates RARβ in Papillary Thyroid Carcinoma. PLoS ONE.

[22] Ramírez-Moya J., Wert-Lamas L., Riesco-Eizaguirre G., Santisteban P. (2019). Impaired microRNA processing by DICER1 downregulation endows thyroid cancer with increased aggressiveness. Oncogene.

[23] Cong D., He M., Chen S., Liu X., Liu X., Sun H. (2015). Expression profiles of pivotal microRNAs and targets in thyroid papillary carcinoma: An analysis of The Cancer Genome Atlas. OncoTargets Ther..

[24] Rosignolo F., Memeo L., Monzani F., Colarossi C., Pecce V., Verrienti A., Durante C., Grani G., Lamartina L., Forte S. (2017). MicroRNA-based molecular classification of papillary thyroid carcinoma. Int. J. Oncol..

[25] Perdas E., Stawski R., Kaczka K., Zubrzycka M. (2020). Analysis of Let-7 Family miRNA in Plasma as Potential Predictive Biomarkers of Diagnosis for Papillary Thyroid Cancer. Diagnostics.

[26] Shabani N., Razaviyan J., Paryan M., Tavangar S.M., Azizi F., Mohammadi-Yeganeh S., Hedayati M. (2018). Evaluation of miRNAs expression in medullary thyroid carcinoma tissue samples: miR-34a and miR-144 as promising overexpressed markers in MTC. Hum. Pathol..

[27] Qiu Z., Li H., Wang J., Sun C. (2017). miR-146a and miR-146b in the diagnosis and prognosis of papillary thyroid carcinoma. Oncol. Rep..

[28] Pamedytyte D., Simanaviciene V., Dauksiene D., Leipute E., Zvirbliene A., Sarauskas V., Dauksa A., Verkauskiene R., Zilaitiene B. (2020). Association of MicroRNA Expression and BRAF^V600E^ Mutation with Recurrence of Thyroid Cancer. Biomolecules.

[29] Rezaei M., Khamaneh A.M., Zarghami N., Vosoughi A., Hashemzadeh S. (2019). Evaluating pre- and post-operation plasma miRNAs of papillary thyroid carcinoma (PTC) patients in comparison to benign nodules. BMC Cancer.

[30] Le F., Luo P., Yang Q.O., Zhong X.M. (2017). MiR-181a promotes growth of thyroid cancer cells by targeting tumor suppressor RB1. Eur. Rev. Med. Pharmacol. Sci..

[31] Liu Y., Zhang B., Shi T., Qin H. (2017). miR-182 promotes tumor growth and increases chemoresistance of human anaplastic thyroid cancer by targeting tripartite motif 8. OncoTargets Ther..

[32] Calabrese G., Dolcimascolo A., Torrisi F., Zappalà A., Gulino R., Parenti R. (2018). MiR-19a Overexpression in FTC-133 Cell Line Induces a More De-Differentiated and Aggressive Phenotype. Int. J. Mol. Sci..

[33] Vosgha H., Ariana A., Smith R.A., Lam A.K. (2018). *miR-205* targets angiogenesis and EMT concurrently in anaplastic thyroid carcinoma. Endocr. Relat. Cancer.

[34] Diao Y., Fu H., Wang Q. (2017). MiR-221 Exacerbate Cell Proliferation and Invasion by Targeting TIMP3 in Papillary Thyroid Carcinoma. Am. J. Ther..

[35] Wei Z.L., Gao A.B., Wang Q., Lou X.E., Zhao J., Lu Q.J. (2019). MicroRNA-221 promotes papillary thyroid carcinoma cell migration and invasion via targeting RECK and regulating epithelial-mesenchymal transition. OncoTargets Ther..

[36] Mazeh H., Deutch T., Karas A., Bogardus K.A., Mizrahi I., Gur-Wahnon D., Ben-Dov I.Z. (2018). Next-Generation Sequencing Identifies a Highly Accurate miRNA Panel That Distinguishes Well-Differentiated Thyroid Cancer from Benign Thyroid Nodules. Cancer Epidemiol. Biomark. Prev..

[37] Zhang Q., Lin L., Li W., Lu G., Li X. (2019). MiR-223 inhibitor suppresses proliferation and induces apoptosis of thyroid cancer cells by down-regulating aquaporin-1. J. Recept. Signal Transduct. Res..

[38] Wen Q., Zhang D., Wang T., Wang T., Zhao M., Xie Q., Fu Y., Ma Q. (2016). MiR-23a promotes cell proliferation and invasion in papillary thyroid carcinoma by targeting PTEN. Int. J. Clin. Exp. Pathol..

[39] Zhao P., Ma W., Hu Z., Zhang Y., Zhang S., Wang Y. (2018). Up-regulation of miR-340-5p promotes progression of thyroid cancer by inhibiting BMP4. J. Endocrinol. Investig..

[40] Chen Y., Zhang S., Zhao R., Zhao Q., Zhang T. (2017). Upregulated miR-9-3p Promotes Cell Growth and Inhibits Apoptosis in Medullary Thyroid Carcinoma by Targeting BLCAP. Oncol. Res..

[41] Mancikova V., Castelblanco E., Pineiro-Yanez E., Perales-Paton J., de Cubas A.A., Inglada-Perez L., Matias-Guiu X., Capel I., Bella M., Lerma E. (2015). MicroRNA deep-sequencing reveals master regulators of follicular and papillary thyroid tumors. Mod. Pathol..

[42] Li H., Zhao L., Zhang Z., Zhang H., Ding C., Su Z. (2017). Roles of microRNA let-7b in papillary thyroid carcinoma by regulating HMGA2. Tumour Biol..

[43] Cao X.Z., Bin H., Zang Z.N. (2019). MiR-128 suppresses the growth of thyroid carcinoma by negatively regulating SPHK1. Biomed. Pharmacother..

[44] Gao X., Chen Z., Li A., Zhang X., Cai X. (2018). MiR-129 regulates growth and invasion by targeting MAL2 in papillary thyroid carcinoma. Biomed. Pharmacother..

[45] Chen X., Li M., Zhou H., Zhang L. (2019). miR-132 Targets FOXA1 and Exerts Tumor-Suppressing Functions in Thyroid Cancer. Oncol. Res..

[46] Montero-Conde C., Graña-Castro O., Martín-Serrano G., Martínez-Montes Á.M., Zarzuela E., Muñoz J., Torres-Perez R., Pita G., Cordero-Barreal A., Leandro-García L.J. (2020). Hsa-miR-139-5p is a prognostic thyroid cancer marker involved in HNRNPF-mediated alternative splicing. Int. J. Cancer..

[47] Sun J., Shi R., Zhao S., Li X., Lu S., Bu H., Ma X., Su C. (2017). E2F8, a direct target of miR-144, promotes papillary thyroid cancer progression via regulating cell cycle. J. Exp. Clin. Cancer Res..

[48] Joo L.J.S., Weiss J., Gill A.J., Clifton-Bligh R., Brahmbhatt H., MacDiarmid J.A., Gild M.L., Robinson B.G., Zhao J.T., Sidhu S.B. (2019). RET Kinase-Regulated MicroRNA-153-3p Improves Therapeutic Efficacy in Medullary Thyroid Carcinoma. Thyroid.

[49] Liu C., Xing M., Wang L., Zhang K. (2017). miR-199a-3p downregulation in thyroid tissues is associated with invasion and metastasis of papillary thyroid carcinoma. Br. J. Biomed. Sci..

[50] Wu X., Dai L., Zhang Z., Zheng J., Zhao J. (2020). Overexpression of microRNA-203 can downregulate survivin and function as a potential therapeutic target in papillary thyroid cancer. Oncol. Lett..

[51] Li D., Wang Q., Li N., Zhang S. (2018). miR-205 targets YAP1 and inhibits proliferation and invasion in thyroid cancer cells. Mol. Med. Rep..

[52] Huang Z., Xing S., Liu M., Deng W., Wang Y., Huang Z., Huang Y., Huang X., Wu C., Guo X. (2019). MiR-26a-5p enhances cells proliferation, invasion, and apoptosis resistance of fibroblast-like synoviocytes in rheumatoid arthritis by regulating PTEN/PI3K/AKT pathway. Biosci. Rep..

[53] Liu Y., Zhang X.L., Li X.F., Tang Y.C., Zhao X. (2018). miR-212-3p reduced proliferation, and promoted apoptosis of fibroblast-like synoviocytes via down-regulating SOX5 in rheumatoid arthritis. Eur. Rev. Med. Pharmacol. Sci..

[54] Lin Y., Cheng K., Wang T., Xie Q., Chen M., Chen Q., Wen Q. (2017). miR-217 inhibits proliferation, migration, and invasion via targeting AKT3 in thyroid cancer. Biomed. Pharmacother..

[55] Yin J.J., Cheng X.Y. (2019). MicroRNA-23a inhibits the growth of papillary thyroid carcinoma via regulating cyclin G1. Eur. Rev. Med. Pharmacol. Sci..

[56] Zhou A., Chen G., Cheng X., Zhang C., Xu H., Qi M., Chen X., Wang T., Li L. (2019). Inhibitory effects of miR-26b-5p on thyroid cancer. Mol. Med. Rep..

[57] Liu H., Deng H., Zhao Y., Li C., Liang Y. (2018). LncRNA XIST/miR-34a axis modulates the cell proliferation and tumor growth of thyroid cancer through MET-PI3K-AKT signaling. J. Exp. Clin. Cancer Res..

[58] Wu G., Zheng H., Xu J., Guo Y., Zheng G., Ma C., Hao S., Liu X., Chen H., Wei S. (2019). miR-429 suppresses cell growth and induces apoptosis of human thyroid cancer cell by targeting ZEB1. Artif. Cells Nanomed. Biotechnol..

[59] Lassalle S., Zangari J., Popa A., Ilie M., Hofman V., Long E., Patey M., Tissier F., Belléannée G., Trouette H. (2016). MicroRNA-375/SEC23A as biomarkers of the in vitro efficacy of vandetanib. Oncotarget.

[60] Xue K.C., Hu D.D., Zhao L., Li N., Shen H.Y. (2017). MiR-577 inhibits papillary thyroid carcinoma cell proliferation, migration and invasion by targeting SphK2. Eur. Rev. Med. Pharmacol. Sci..

[61] Gu Y., Yang N., Yin L., Feng C., Liu T. (2018). Inhibitory roles of miR-9 on papillary thyroid cancer through targeting BRAF. Mol. Med. Rep..

[62] Ding C., Yu H., Shi C., Shi T., Qin H., Cui Y. (2019). MiR-let-7e inhibits invasion and magration and regulates HMGB1 expression in papillary thyroid carcinoma. Biomed. Pharmacother..

[63] Ahmed R., Samanta S., Banerjee J., Kar S.S., Dash S.K. (2022). Modulatory role of miRNAs in thyroid and breast cancer progression and insights into their therapeutic manipulation. Curr. Res. Pharmacol. Drug Discov..

[64] Lu M.D., Li H., Nie J.H., Li S., Ye H.S., Li T.T., Wu M.L., Liu J. (2022). Dual Inhibition of BRAF-MAPK and STAT3 Signaling Pathways in Resveratrol-Suppressed Anaplastic Thyroid Cancer Cells with BRAF Mutations. Int. J. Mol. Sci..

[65] Zhang C., Gu H., Liu D., Tong F., Wei H., Zhou D., Fang J., Dai X., Tian H. (2022). The circ_FAM53B-miR-183-5p-CCDC6 axis modulates the malignant behaviors of papillary thyroid carcinoma cells. Mol. Cell. Biochem..

[66] Zhang K., Liu J., Li C., Peng X., Li H., Li Z. (2019). Identification and validation of potential target genes in papillary thyroid cancer. Eur. J. Pharmacol..

[67] Barkovskaya A., Buffone A., Žídek M., Weaver V.M. (2020). Proteoglycans as Mediators of Cancer Tissue Mechanics. Front. Cell Dev. Biol..

[68] Vasioukhin V. (2012). Adherens junctions and cancer. Subcell. Biochem..

[69] Long M., Zhu Y., Chen Z., Lin S., Peng X., Luo D., Li H., Tan L. (2020). Lysine-Specific Demethylase 1 Affects the Progression of Papillary Thyroid Carcinoma via HIF1α and microRNA-146a. J. Clin. Endocrinol. Metab..

[70] Mostafaei S., Keshavarz M., Sadri Nahand J., Farhadi Hassankiadeh R., Moradinazar M., Nouri M., Babaei F., Ahadi M., Payandeh M., Salari Esker A. (2020). Viral infections and risk of thyroid cancer: A systematic review and empirical bayesian meta-analysis. Pathol. Res. Pract..

[71] Ignjatović V., Janković Miljuš J.R., Rončević J.V., Tatić S.B., Išić Denčić T.M., Đorić I.Đ., Šelemetjev S.A. (2022). Focal adhesion kinase splicing and protein activation in papillary thyroid carcinoma progression. Histochem. Cell Biol..

[72] Hossain M.A., Asa T.A., Rahman M.M., Uddin S., Moustafa A.A., Quinn J.M.W., Moni M.A. (2020). Network-Based Genetic Profiling Reveals Cellular Pathway Differences Between Follicular Thyroid Carcinoma and Follicular Thyroid Adenoma. Int. J. Environ. Res. Public Health.

[73] Xie J., Liu Y., Du X., Wu Y. (2019). TGF-β1 promotes the invasion and migration of papillary thyroid carcinoma cells by inhibiting the expression of lncRNA-NEF. Oncol. Lett..

[74] Fazekas-Lavu M., Parker A., Spigelman A.D., Scott R.J., Epstein R.J., Jensen M., Samaras K. (2017). Thyroid cancer in a patient with Lynch syndrome—Case report and literature review. Ther. Clin. Risk Manag..

[75] Nieminen T.T., Walker C.J., Olkinuora A., Genutis L.K., O'Malley M., Wakely P.E., LaGuardia L., Koskenvuo L., Arola J., Lepistö A.H. (2020). Thyroid Carcinomas That Occur in Familial Adenomatous Polyposis Patients Recurrently Harbor Somatic Variants in *APC*, *BRAF*, and *KTM2D*. Thyroid.

[76] Ciavardelli D., Bellomo M., Consalvo A., Crescimanno C., Vella V. (2017). Metabolic Alterations of Thyroid Cancer as Potential Therapeutic Targets. Biomed. Res. Int..

[77] Qin Y., Sun W., Wang Z., Dong W., He L., Zhang T., Lv C., Zhang H. (2022). RBM47/SNHG5/FOXO3 axis activates autophagy and inhibits cell proliferation in papillary thyroid carcinoma. Cell Death Dis..

[78] Huo N., Cong R., Sun Z.J., Li W.C., Zhu X., Xue C.Y., Chen Z., Ma L.Y., Chu Z., Han Y.C. (2021). STAT3/LINC00671 axis regulates papillary thyroid tumor growth and metastasis via LDHA-mediated glycolysis. Cell Death Dis..

[79] Wang L., Zhang W.P., Yao L., Zhang W., Zhu J., Zhang W.C., Zhang Y.H., Wang Z., Yan Q.G., Guo Y. (2015). PRDM1 expression via human parvovirus B19 infection plays a role in the pathogenesis of Hashimoto thyroiditis. Hum. Pathol..

[80] Colombo C., Pogliaghi G., Tosi D., Muzza M., Bulfamante G., Persani L., Fugazzola L., Cirello V. (2022). Thyroid cancer harboring *PTEN* and *TP53* mutations: A peculiar molecular and clinical case report. Front. Oncol..

[81] Sawicka-Gutaj N., Shawkat S., Andrusiewicz M., Ziółkowska P., Czarnywojtek A., Gut P., Ruchała M. (2021). *EZH2* and *SMYD3* expression in papillary thyroid cancer. Oncol. Lett..

[82] Plantinga T.S., Petrulea M.S., Oosting M., Joosten L.A.B., Piciu D., Smit J.W., Netea-Maier R.T., Georgescu C.E. (2017). Association of NF-κB polymorphisms with clinical outcome of non-medullary thyroid carcinoma. Endocr. Relat. Cancer.

[83] Crezee T., Petrulea M., Piciu D., Jaeger M., Smit J.W.A., Plantinga T.S., Georgescu C.E., Netea-Maier R. (2020). Akt1 genetic variants confer increased susceptibility to thyroid cancer. Endocr. Connect..

[84] Gong Z., Yang S., Wei M., Vlantis A.C., Chan J.Y.K., van Hasselt C.A., Li D., Zeng X., Xue L., Tong M.C.F. (2022). The Isoforms of Estrogen Receptor α and β in Thyroid Cancer. Front. Oncol..

[85] Asya O., Yumuşakhuylu A.C., Bağcı P., Kaya H., Gönen A., Gündoğdu Y., Muradov T., Şahin A., Oysu Ç. (2022). Relationship of *PPARG* overexpression with prognostic parameters in papillary thyroid carcinoma. Acta. Otorhinolaryngol. Ital..

[86] Kovaleva I.V., Spirina L.V., Chizhevskaya S.Y., Kondakova I.V., Choinzonov E.L. (2022). Expression and content of LC3B protein in tissues of papillary thyroid cancer, relationship with clinical and morphological parameters of tumors. Probl. Oncol..

[87] Humbert M., Morán M., de la Cruz-Ojeda P., Muntané J., Wiedmer T., Apostolova N., McKenna S.L., Velasco G., Balduini W., Eckhart L. (2020). Assessing Autophagy in Archived Tissue or How to Capture Autophagic Flux from a Tissue Snapshot. Biology.

