# Supplementary material for: Autophagy-Related MicroRNA: Tumor miR-125b and Thyroid Cancers
Source: Genes (Basel). 2023 Mar 9;14(3):685. doi: 10.3390/genes14030685 (PMC10048312; doi:10.3390/genes14030685)
Supplement: Supplementary file 1 [file genes-14-00685-s001.zip › genes-2235398-supplementary.pdf]

Table S1. List of microRNAs associated with the autophagy-related proteins.

Protein	microRNA
ATG12	hsa-let-7a-5p
	hsa-miR-124-3p
	hsa-miR-1301-3p
	hsa-miR-1304-5p
	hsa-miR-1-3p
	hsa-miR-147a
	hsa-miR-16-2-3p
	hsa-miR-17-3p
	hsa-miR-191-5p
	hsa-miR-200b-3p
	hsa-miR-200c-3p
	hsa-miR-203a-3p
	hsa-miR-30a-5p
	hsa-miR-30b-5p
	hsa-miR-30c-5p
	hsa-miR-30d-5p
	hsa-miR-30e-5p
	hsa-miR-376a-5p
	hsa-miR-424-5p
	hsa-miR-495-3p
	hsa-miR-522-5p
	hsa-miR-577
ATG14	hsa-miR-10b-5p
	hsa-miR-124-3p
	hsa-miR-130a-3p

ATG16L1	hsa-miR-1-3p<br>hsa-miR-146a-5p<br>hsa-miR-155-5p<br>hsa-miR-15a-5p<br>hsa-miR-16-5p<br>hsa-miR-191-5p<br>hsa-miR-195-5p<br>hsa-miR-19b-3p<br>hsa-miR-210-3p<br>hsa-miR-212-3p<br>hsa-miR-221-3p<br>hsa-miR-224-5p<br>hsa-miR-23a-3p<br>hsa-miR-23b-3p<br>hsa-miR-26a-5p<br>hsa-miR-34a-5p<br>hsa-miR-424-5p<br>hsa-miR-588<br>hsa-miR-616-5p<br>hsa-miR-9-5p<br>hsa-let-7a-5p<br>hsa-let-7b-5p<br>hsa-let-7c-5p<br>hsa-let-7e-5p<br>hsa-let-7f-5p<br>hsa-let-7g-5p<br>hsa-let-7i-5p

hsa-miR-103a-3p
hsa-miR-106a-5p
hsa-miR-106b-5p
hsa-miR-107
hsa-miR-1179
hsa-miR-1207-5p
hsa-miR-1234-3p
hsa-miR-124-3p
hsa-miR-126-5p
hsa-miR-128-3p
hsa-miR-1306-5p
hsa-miR-130a-3p
hsa-miR-130b-3p
hsa-miR-1343-3p
hsa-miR-1-3p
hsa-miR-141-3p
hsa-miR-147b
hsa-miR-148a-3p
hsa-miR-148a-5p
hsa-miR-148b-3p
hsa-miR-148b-5p
hsa-miR-155-5p
hsa-miR-17-5p
hsa-miR-181c-3p
hsa-miR-185-3p
hsa-miR-1914-3p

		hsa-miR-191-5p
		hsa-miR-196a-5p
		hsa-miR-196b-5p
		hsa-miR-19a-3p
		hsa-miR-19b-3p
		hsa-miR-200a-3p
		hsa-miR-200b-3p
		hsa-miR-20a-5p
		hsa-miR-20b-5p
		hsa-miR-212-5p
		hsa-miR-216a-5p
		hsa-miR-224-5p
		hsa-miR-26a-5p
		hsa-miR-26b-5p
		hsa-miR-27a-5p
		hsa-miR-29a-3p
		hsa-miR-29a-5p
		hsa-miR-29b-3p
		hsa-miR-29c-3p
		hsa-miR-301a-3p
		hsa-miR-301b-3p
		hsa-miR-30a-3p
		hsa-miR-30d-3p
		hsa-miR-30e-3p
		hsa-miR-3187-3p
		hsa-miR-331-3p

	hsa-miR-331-5p
	hsa-miR-338-3p
	hsa-miR-3934-5p
	hsa-miR-423-5p
	hsa-miR-452-3p
	hsa-miR-452-5p
	hsa-miR-454-3p
	hsa-miR-494-3p
	hsa-miR-5008-3p
	hsa-miR-522-5p
	hsa-miR-588
	hsa-miR-652-3p
	hsa-miR-7-5p
	hsa-miR-93-5p
	hsa-miR-941
	hsa-miR-98-5p
	kshv-miR-K12-10a-3p
ATG5	hsa-miR-124-3p
	hsa-miR-125a-3p
	hsa-miR-135b-5p
	hsa-miR-1-3p
	hsa-miR-153-3p
	hsa-miR-155-5p
	hsa-miR-16-5p
	hsa-miR-182-5p
	hsa-miR-186-5p
	hsa-miR-19a-3p

	hsa-miR-19b-3p
	hsa-miR-210-3p
	hsa-miR-24-3p
	hsa-miR-25-3p
	hsa-miR-30d-5p
	hsa-miR-32-5p
	hsa-miR-342-3p
	hsa-miR-377-5p
	hsa-miR-429
	hsa-miR-548b-3p
	hsa-miR-5581-3p
	hsa-miR-92a-3p
BECN1	hsa-miR-101-5p
	hsa-miR-103a-3p
	hsa-miR-107
	hsa-miR-1224-5p
	hsa-miR-124-3p
	hsa-miR-130a-3p
	hsa-miR-130b-3p
	hsa-miR-1-3p
	hsa-miR-147a
	hsa-miR-15a-5p
	hsa-miR-15b-5p
	hsa-miR-16-5p
	hsa-miR-183-5p
	hsa-miR-186-5p
	hsa-miR-191-5p

	hsa-miR-195-5p
	hsa-miR-224-5p
	hsa-miR-28-5p
	hsa-miR-301a-3p
	hsa-miR-301b-3p
	hsa-miR-302a-3p
	hsa-miR-302b-3p
	hsa-miR-302c-3p
	hsa-miR-302d-3p
	hsa-miR-30a-3p
	hsa-miR-30a-5p
	hsa-miR-30b-5p
	hsa-miR-30c-5p
	hsa-miR-30d-5p
	hsa-miR-30e-3p
	hsa-miR-30e-5p
	hsa-miR-320a
	hsa-miR-323a-5p
	hsa-miR-328-3p
	hsa-miR-424-5p
	hsa-miR-505-5p
	hsa-miR-522-5p
	hsa-miR-548b-3p
	hsa-miR-550a-3p
	hsa-miR-708-5p
MAP1LC3	hsa-let-7g-3p
	hsa-miR-106b-5p

	hsa-miR-124-3p
	hsa-miR-129-2-3p
	hsa-miR-129-5p
	hsa-miR-132-3p
	hsa-miR-1-3p
	hsa-miR-145-5p
	hsa-miR-155-5p
	hsa-miR-16-5p
	hsa-miR-191-5p
	hsa-miR-192-5p
	hsa-miR-200b-3p
	hsa-miR-20a-5p
	hsa-miR-21-3p
	hsa-miR-218-5p
	hsa-miR-23b-3p
	hsa-miR-27a-5p
	hsa-miR-29a-5p
	hsa-miR-30a-5p
	hsa-miR-30c-2-3p
	hsa-miR-335-5p
	hsa-miR-433-3p
	hsa-miR-4738-3p
	hsa-miR-483-3p
	hsa-miR-485-3p
	hsa-miR-495-3p
	hsa-miR-574-5p

		hsa-miR-760
		hsa-miR-9-5p
		hsa-let-7a-3p
		hsa-let-7a-5p
		hsa-let-7b-5p
		hsa-let-7c-5p
		hsa-let-7f-1-3p
		hsa-let-7f-5p
		hsa-let-7g-5p
		hsa-let-7i-5p
		hsa-miR-106a-5p
		hsa-miR-106b-5p
		hsa-miR-1226-3p
		hsa-miR-124-3p
	ULK1	hsa-miR-1254
		hsa-miR-1260a
		hsa-miR-1287-5p
		hsa-miR-129-2-3p
		hsa-miR-1293
		hsa-miR-1301-3p
		hsa-miR-132-5p
		hsa-miR-1343-3p
		hsa-miR-1-3p
		hsa-miR-142-5p
		hsa-miR-146a-5p
		hsa-miR-146b-5p
		hsa-miR-147a

hsa-miR-155-5p
hsa-miR-16-5p
hsa-miR-17-5p
hsa-miR-181a-5p
hsa-miR-181b-5p
hsa-miR-183-5p
hsa-miR-186-5p
hsa-miR-18a-3p
hsa-miR-18a-5p
hsa-miR-1908-5p
hsa-miR-1910-5p
hsa-miR-19a-3p
hsa-miR-200b-3p
hsa-miR-203a-3p
hsa-miR-20a-5p
hsa-miR-20b-5p
hsa-miR-2114-5p
hsa-miR-21-3p
hsa-miR-221-5p
hsa-miR-22-3p
hsa-miR-23a-3p
hsa-miR-23b-3p
hsa-miR-26a-5p
hsa-miR-26b-5p
hsa-miR-27a-5p
hsa-miR-296-5p

	hsa-miR-29a-3p
	hsa-miR-29b-3p
	hsa-miR-29c-3p
	hsa-miR-3065-3p
	hsa-miR-30b-3p
	hsa-miR-30c-1-3p
	hsa-miR-30c-2-3p
	hsa-miR-320a
	hsa-miR-320b
	hsa-miR-320c
	hsa-miR-320d
	hsa-miR-32-5p
	hsa-miR-331-3p
	hsa-miR-335-5p
	hsa-miR-338-3p
	hsa-miR-338-5p
	hsa-miR-33b-3p
	hsa-miR-33b-5p
	hsa-miR-342-5p
	hsa-miR-34a-5p
	hsa-miR-34c-5p
	hsa-miR-361-3p
	hsa-miR-3661
	hsa-miR-378a-3p
	hsa-miR-378c
	hsa-miR-378d

	hsa-miR-378i
	hsa-miR-3943
	hsa-miR-421
	hsa-miR-424-5p
	hsa-miR-4487
	hsa-miR-4804-5p
	hsa-miR-484
	hsa-miR-489-3p
	hsa-miR-490-5p
	hsa-miR-497-5p
	hsa-miR-5008-5p
	hsa-miR-503-5p
	hsa-miR-505-5p
	hsa-miR-5690
	hsa-miR-589-5p
	hsa-miR-590-5p
	hsa-miR-615-5p
	hsa-miR-642a-5p
	hsa-miR-744-5p
	hsa-miR-760
	hsa-miR-769-3p
	hsa-miR-873-5p
	hsa-miR-92a-3p
	hsa-miR-92b-3p
	hsa-miR-93-3p
	hsa-miR-93-5p

	hsa-miR-9-3p
	hsa-miR-940
MTOR	hsa-miR-99b-5p
	hsa-let-7b-5p
	hsa-let-7d-5p
	hsa-miR-100-5p
	hsa-miR-122-5p
	hsa-miR-124-3p
	hsa-miR-1343-3p
	hsa-miR-144-3p
	hsa-miR-16-2-3p
	hsa-miR-16-5p
	hsa-miR-17-5p
	hsa-miR-191-5p
	hsa-miR-199a-3p
	hsa-miR-20a-3p
	hsa-miR-20a-5p
	hsa-miR-21-3p
	hsa-miR-218-5p
	hsa-miR-221-3p
	hsa-miR-223-3p
	hsa-miR-27a-3p
	hsa-miR-30a-5p
	hsa-miR-3657
	hsa-miR-373-3p
	hsa-miR-382-5p
	hsa-miR-421
	hsa-miR-424-5p
	hsa-miR-4425
	hsa-miR-497-5p
	hsa-miR-520c-3p
	hsa-miR-548o-3p
	hsa-miR-616-5p
	hsa-miR-766-3p
	hsa-miR-95-3p
	hsa-miR-99a-5p
	hsa-let-7a-3p
PRKAA1	hsa-let-7a-5p
	hsa-let-7b-5p
	hsa-let-7c-5p
	hsa-let-7e-5p
	hsa-let-7f-1-3p
	hsa-let-7f-5p

hsa-let-7g-3p
hsa-let-7g-5p
hsa-let-7i-5p
hsa-miR-103a-3p
hsa-miR-106b-5p
hsa-miR-107
hsa-miR-10a-3p
hsa-miR-122-5p
hsa-miR-124-3p
hsa-miR-126-5p
hsa-miR-1276
hsa-miR-128-3p
hsa-miR-1290
hsa-miR-1301-3p
hsa-miR-1304-3p
hsa-miR-1306-5p
hsa-miR-130a-3p
hsa-miR-130b-3p
hsa-miR-133a-3p
hsa-miR-1-3p
hsa-miR-141-3p
hsa-miR-141-5p
hsa-miR-146a-5p
hsa-miR-146b-5p
hsa-miR-147a
hsa-miR-148a-3p

	hsa-miR-148b-3p
	hsa-miR-152-3p
	hsa-miR-182-5p
	hsa-miR-188-5p
	hsa-miR-194-5p
	hsa-miR-19a-3p
	hsa-miR-19a-5p
	hsa-miR-19b-1-5p
	hsa-miR-19b-3p
	hsa-miR-200a-3p
	hsa-miR-205-3p
	hsa-miR-20a-5p
	hsa-miR-216b-5p
	hsa-miR-217
	hsa-miR-218-5p
	hsa-miR-224-3p
	hsa-miR-224-5p
	hsa-miR-23a-3p
	hsa-miR-23b-3p
	hsa-miR-26a-5p
	hsa-miR-27a-3p
	hsa-miR-27b-3p
	hsa-miR-28-5p
	hsa-miR-29a-3p
	hsa-miR-29b-3p
	hsa-miR-29c-3p

hsa-miR-301a-3p
hsa-miR-301b-3p
hsa-miR-30a-3p
hsa-miR-30a-5p
hsa-miR-30d-3p
hsa-miR-30e-3p
hsa-miR-3145-3p
hsa-miR-3168
hsa-miR-320a
hsa-miR-320b
hsa-miR-320c
hsa-miR-320d
hsa-miR-328-3p
hsa-miR-335-3p
hsa-miR-339-5p
hsa-miR-33b-5p
hsa-miR-340-5p
hsa-miR-361-5p
hsa-miR-3619-5p
hsa-miR-373-3p
hsa-miR-374a-3p
hsa-miR-374a-5p
hsa-miR-421
hsa-miR-425-5p
hsa-miR-4326
hsa-miR-4454
hsa-miR-451a

	hsa-miR-454-3p
	hsa-miR-4687-5p
	hsa-miR-4690-5p
	hsa-miR-483-3p
	hsa-miR-499a-5p
	hsa-miR-5008-3p
	hsa-miR-500a-3p
	hsa-miR-500a-5p
	hsa-miR-505-3p
	hsa-miR-548a-3p
	hsa-miR-548e-3p
	hsa-miR-5581-5p
	hsa-miR-571
	hsa-miR-579-3p
	hsa-miR-589-5p
	hsa-miR-590-3p
	hsa-miR-605-5p
	hsa-miR-617
	hsa-miR-628-5p
	hsa-miR-664b-3p
	hsa-miR-708-5p
	hsa-miR-7-1-3p
	hsa-miR-7-5p
	hsa-miR-769-3p
	hsa-miR-93-3p
	hsa-miR-93-5p

	hsa-miR-941
	hsa-miR-96-5p
	hsa-miR-98-5p
	hsa-miR-99b-3p
